# Pneumococcal Capsular Polysaccharide Structure Predicts Serotype Prevalence

**DOI:** 10.1371/journal.ppat.1000476

**Published:** 2009-06-12

**Authors:** Daniel M. Weinberger, Krzysztof Trzciński, Ying-Jie Lu, Debby Bogaert, Aaron Brandes, James Galagan, Porter W. Anderson, Richard Malley, Marc Lipsitch

**Affiliations:** 1 Department of Immunology and Infectious Diseases, Harvard School of Public Health, Boston, Massachusetts, United States of America; 2 Department of Epidemiology, Harvard School of Public Health, Boston, Massachusetts, United States of America; 3 Division of Infectious Diseases, Department of Medicine, Children's Hospital and Harvard Medical School, Boston, Massachusetts, United States of America; 4 Broad Institute of MIT and Harvard, Cambridge, Massachusetts, United States of America; Johns Hopkins School of Medicine, United States of America

## Abstract

There are 91 known capsular serotypes of *Streptococcus pneumoniae*. The nasopharyngeal carriage prevalence of particular serotypes is relatively stable worldwide, but the host and bacterial factors that maintain these patterns are poorly understood. Given the possibility of serotype replacement following vaccination against seven clinically important serotypes, it is increasingly important to understand these factors. We hypothesized that the biochemical structure of the capsular polysaccharides could influence the degree of encapsulation of different serotypes, their susceptibility to killing by neutrophils, and ultimately their success during nasopharyngeal carriage. We sought to measure biological differences among capsular serotypes that may account for epidemiological patterns. Using an *in vitro* assay with both isogenic capsule-switch variants and clinical carriage isolates, we found an association between increased carriage prevalence and resistance to non-opsonic neutrophil-mediated killing, and serotypes that were resistant to neutrophil-mediated killing tended to be more heavily encapsulated, as determined by FITC-dextran exclusion. Next, we identified a link between polysaccharide structure and carriage prevalence. Significantly, non-vaccine serotypes that have become common in vaccinated populations tend to be those with fewer carbons per repeat unit and low energy expended per repeat unit, suggesting a novel biological principle to explain patterns of serotype replacement. More prevalent serotypes are more heavily encapsulated and more resistant to neutrophil-mediated killing, and these phenotypes are associated with the structure of the capsular polysaccharide, suggesting a direct relationship between polysaccharide biochemistry and the success of a serotype during nasopharyngeal carriage and potentially providing a method for predicting serotype replacement.

## Author Summary

*Streptococcus pneumoniae*, or pneumococcus, is an important pathogen worldwide and causes a wide range of diseases, mostly in young children and the elderly. There are 91 serotypes of pneumococcus, each of which produces a unique polysaccharide, called the capsule, that attaches to the bacterial surface and prevents it from being cleared by the host. The serotypes differ greatly in their prevalence in the human population. There is currently a vaccine, effective in infancy, which targets seven clinically important serotypes, but several types not covered by the vaccine are beginning to increase in carriage frequency. As a result, it is critical to understand why some serotypes are frequently carried in the human population while others are not. In this study, we find that the high-prevalence serotypes tend to be more heavily encapsulated and more resistant to killing by neutrophils. Significantly, we find that the biochemical properties of the different polysaccharides can be used to predict their carriage frequency both before and after introduction of the vaccine. These results provide a biologically plausible explanation for differences in prevalence between serotypes.

## Introduction

*Streptococcus pneumoniae*, or pneumococcus, is an important pathogen worldwide and is a causative agent of pneumonia, meningitis and otitis media. There are 91 known pneumococcal serotypes, and each produces a biochemically distinct polysaccharide capsule that is in most cases covalently attached to the cell wall. Serotype affects nearly every aspect of pneumococcal pathogenesis and of nasopharyngeal carriage, which precedes disease and serves as the reservoir for transmission of the organism [1]. The serotypes most common in both invasive disease [2],[3] and carriage [4] show remarkable consistency across geography and time despite some differences in the details. Serotypes differ not only in their prevalence, but also in their tendency to cause invasive or mucosal disease (ratio of disease cases to carriers) [5]–[8], their age distribution [4], their tendency to cause outbreaks [4],[9], and their degree of antimicrobial resistance [10].

The polysaccharide-protein pneumococcal conjugate vaccine (Prevnar, or PCV7) targets seven clinically relevant serotypes in young children. Currently, the vaccine is in widespread use, and while PCV7 has been largely successful at reducing the burden of invasive pneumococcal disease in the United States. [11], evidence is emerging that serotypes not targeted by the vaccine, such as types 19A and 15, are increasing in importance in both carriage and invasive disease [12]–[15]. In the long-term, disease caused by replacement serotypes could partially undermine the impact of the vaccine. As more countries introduce PCV7 or explore alternative polysaccharide formulations, it will be critical to understand the factors that determine serotype patterns of carriage. We sought to measure the biological differences among capsular serotypes that may account for these effects on pneumococcal epidemiology and then use these biological measures as predictors of prevalence before and after vaccination.

Duration of carriage, a determinant of prevalence, varies between serotypes [16],[17] and could, in part, be influenced by the interactions between bacteria and host immune effectors. The capsule protects against phagocytic clearance by blocking the deposition and function of opsonins directed against cell surface antigens [18]–[20]. In addition, capsule can affect susceptibility to trapping by neutrophil extracellular traps (NETs) [21], killing by defensins [22], and clearance by mucus [23]. Strains that produce more capsule *in vitro* are more virulent *in vivo* [24], but degree of encapsulation has not previously been shown to strongly impact nasopharyngeal colonization [25].

Immune-mediated clearance from the nasopharynx involves both antibody-dependent and antibody-independent mechanisms of immunity [26]–[29]. Antibody-independent clearance is thought to involve an IL-17A-mediated T-cell response [30],[31], which results in the recruitment of neutrophils to the site of infection and subsequent clearance of colonization [31],[32]. Neutrophils can kill pneumococci in the presence or absence of opsonins, and heavily encapsulated strains can avoid phagocytic uptake [33]–[36].

Capsular polysaccharide quantity and degree of encapsulation could be influenced by a number of factors, and recent work has demonstrated that sugar metabolism could play a regulatory role [37],[38]. We hypothesized that serotypes that require more energy or carbon to synthesize a polysaccharide repeat unit would ultimately have smaller, less inhibitory capsules. In this study, we demonstrate an association between polysaccharide structure, degree of encapsulation, susceptibility to neutrophil-mediated killing, and carriage prevalence. We propose a model in which serotypes that produce metabolically inexpensive polysaccharides will be more heavily encapsulated, which in turn allows them to persist in the nasopharynx for a longer duration and results in higher prevalence. These results will be particularly useful in predicting the impact of serotype-replacement in various settings.

## Results

### Capsule protects against non-opsonic killing by human neutrophils

**Figure 1:**
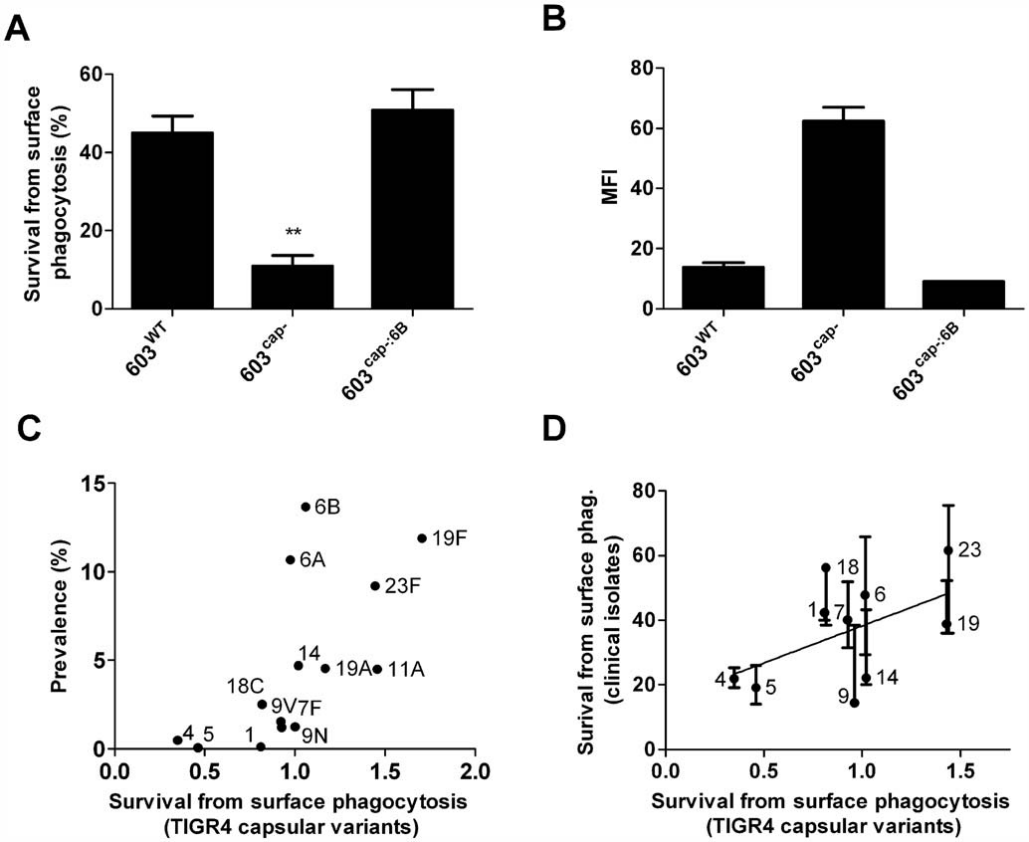
Effect of serotype on resistance to non-opsonic killing by neutrophils. A) Capsule increases resistance of pneumococcus type 6B to non-opsonic surface killing compared to an unencapsulated derivative. Mean+/−SEM from a representative experiment. p<0.001 B) Capsule prevents association of pneumococcus type 6B with human PMNs. Mean+/−range from two independent experiments. MFI: Mean fluorescence intensity of neutrophils. C) Avoidance of neutrophil-mediated killing by the TIGR4 capsule variants is directly correlated with the frequency of each serotype among carriage isolates. Mean survival relative to serotype 9N is shown. ρ = 0.78, p<0.01. D) Susceptibility to neutrophil-mediated killing is a property of capsular type across diverse genetic backgrounds. Linear regression is shown comparing survival from neutrophil-mediated killing between isogenic TIGR4 capsule variants and diverse clinical isolates. Mean+/−range., ρ = 0.49, p<0.05.

First, we evaluated whether the production of a capsule affected susceptibility to opsonin-independent killing by human neutrophils. We tested an invasive type 6B clinical isolate, its unencapsulated isogenic derivative and the reconstituted strain with the type 6B capsule locus reinserted. The wild type and the reconstituted encapsulated strain were significantly more resistant to killing than the unencapsulated mutant (Figure 1A). Additionally, by flow cytometry we found that the unencapsulated strain was more efficiently associated with neutrophils than the wild type or the reconstituted strain (Figure 1B).

### Resistance to killing correlates with higher carriage prevalence

To test whether highly prevalent serotypes are more resistant to neutrophil-mediated killing, we used a panel of TIGR4 capsule-switch variants that are isogenic except for the capsule locus. The more prevalent serotypes, such as 19F and 23F, were indeed most resistant to killing, while types that are rarely isolated from carriage, such as types 4 and 5, were more efficiently killed (r = 0.77, p<0.001; Figure 1C).

To confirm these results, we tested another set of five isogenic capsule-switch variants that were constructed in strain 603, a type 6B clinical isolate. Again, we found that resistance to neutrophil-mediated killing was associated with higher carriage prevalence (Figure S1). The serotype rank-order of susceptibility to killing was the same in both sets of isogenic capsule-switch variants with the exception of type 6B, which was more resistant to killing in the 603 genetic background.

### Capsule type affects resistance to killing in diverse genetic backgrounds

To determine whether the effect of serotype on avoidance of neutrophil-mediated killing could be generalized to clinical carriage isolates, we tested a set of strains from diverse bacterial genetic backgrounds. There was a significant association between susceptibility to killing of the TIGR4 isogenic capsule variants and the corresponding clinical strain of the same serogroup (Figure 1D). These results indicate that serotype is a major determinant of resistance to neutrophil-mediated killing in diverse genetic backgrounds, though it is not the only determining factor.

### Resistance to killing is related to degree of encapsulation

**Figure 2:**
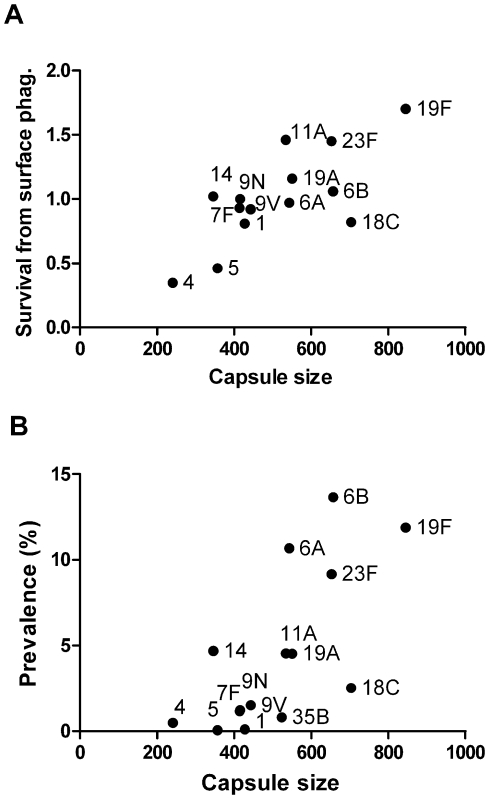
Relationship between degree of encapsulation, resistance to neutrophil-mediated killing, and carriage prevalence. Capsule size, as evaluated by FITC-Dextran exclusion, is related to A) avoidance of neutrophil-mediated killing (ρ = 0.58, p<0.05) and B) carriage prevalence (ρ = 0.68, p<0.01). The mean area of the zone of FITC-dextran exclusion, including the bacterial body and capsule, is shown. 100–250 cells per serotype were measured, and the zone of exclusion for unencapsulated TIGR4 was 286 pixels.

We next wanted to evaluate whether differences in degree of encapsulation between serotypes could affect interactions with neutrophils. To determine the degree of encapsulation, we measured the zone of exclusion of fluorescent dextran molecules by the capsule [39] using our isogenic TIGR4 capsule-switch variants. Serotype significantly influenced the size of the zone of dextran exclusion, and types with larger zones of exclusion were more resistant to neutrophil-mediated killing and more prevalent in carriage (Figure 2). Serotypes that had large zones of dextran exclusion also had clearly visible capsules when suspended in India ink (Figure S2). We also found that pre-incubating the neutrophils with purified capsular polysaccharides had no effect on neutrophil-mediated killing (data not shown), further suggesting that the capsule is blocking the bacterial surface from the neutrophils rather than being itself a target.

**Figure 3:**
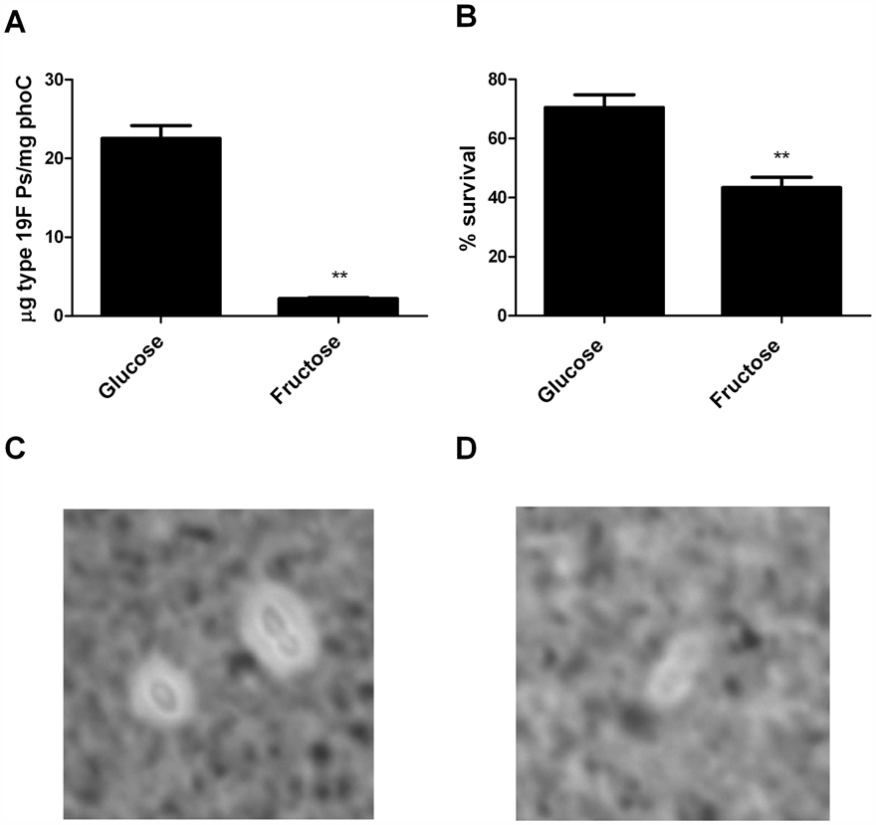
Effect of degree of encapsulation on susceptibility to neutrophil-mediated killing of type 19F. Growth in fructose leads to A) a decrease in cell-associated type 19F polysaccharide as evaluated by inhibition ELISA and B) decreased resistance to neutrophil-mediated killing. Mean+/−SEM, p<0.001. The capsules of bacteria grown in C) glucose or D) fructose were visualized in India ink.

These correlations suggest that degree of encapsulation affects resistance to neutrophil-mediated killing. However, given the differences in structure between polysaccharides, it is possible that some other structural feature, which happens to be correlated with degree of encapsulation, is actually the mechanism of differential resistance to killing. To test the hypothesis that degree of encapsulation is the relevant measure, we made comparisons within a serotype, where structure should be conserved but degree of encapsulation could be manipulated by changing carbon sources, as previously reported [40],[41]. A derivative of TIGR4 producing a type 19F capsule was grown in a semi-defined medium with either fructose, which has been reported to reduce capsule production [41], or glucose. We found that bacteria grown in fructose were less encapsulated than those grown in glucose, both by India Ink microscopy (Figure 3C,D) and by capsular polysaccharide inhibition ELISA (Figure 3A). Significantly, fructose-grown bacteria were more susceptible to surface killing, and heavily encapsulated serotypes were more strongly affected by growth in fructose (Figures 3B, S3). These results, which are consistent with findings in group A *Streptococcus* [42], indicate that degree of encapsulation directly affects susceptibility to non-opsonic neutrophil-mediated killing.

### Serotype affects colonization *in vivo*

To determine whether our *in vitro* findings were applicable to an *in vivo* situation, we co-colonized mice with a mix of isogenic TIGR4 capsule-switch variants producing type 14 and type 19F polysaccharides. These serotypes were chosen because of their different phenotypes in the *in vitro* assays and because they are easily distinguishable by colony morphology on blood agar plates. In past experiments, we have shown that both strains can colonize mice at similar densities when inoculated alone. Based on our *in vitro* results, we hypothesized that type 19F would out-compete type 14.

We intranasally inoculated mice with a ratio of 100 CFU of type 14 to 1 CFU of type 19F (proportion 19F = 0.01). Consistent with our hypothesis, while only 1% of the inoculum was type 19F, 90% of the colonies recovered from the nasal washes after 7 days were type 19F, indicating that type 19F colonizes mice significantly better than type 14 (4 mice, average proportion 19F: 0.90, SEM: 0.04, binomial probability: p<0.001 compared to inoculum).

### Polysaccharide structure is associated with prevalence, degree of encapsulation, and neutrophil-mediated killing

Having found a relationship between prevalence and degree of encapsulation and resistance to neutrophil-mediated killing, we next wanted to evaluate bacterial factors that could influence these phenotypes. We hypothesized that the extent of encapsulation, and subsequently the epidemiologic properties of the serotype, could be constrained by the metabolic requirements for biosynthesis of different capsular polysaccharides. By examining published polysaccharide structures and biochemical pathways, we determined the number of carbons and the number of high-energy bonds (ATP-equivalents) that are required to generate one polysaccharide repeat unit. Consistent with the hypothesis, we found a significant association between these measures of metabolic cost and degree of encapsulation and a trend between metabolic cost and resistance to non-opsonic killing (Table 1).

**Table 1:** Relationship between capsular polysaccharide composition, degree of encapsulation, neutrophil-mediated killing, and prevalence.

	Carbon	High energy
**Capsule Size**	−0.60^†^	−0.70^†^
**Resistance to neutrophil killing**	−0.48	−0.51
**Pre-PCV7 frequency**	−0.73^†^	−0.72^†^
**Pre-PCV7 frequency (excl type 3)**	−0.80^††^	−0.79^††^
**Post-PCV7 frequency**	−0.40	−0.31
**Post-PCV7 frequency (excl type 3)**	−0.90^†^	−0.88^†^

The number of carbons or the number of high energy molecules consumed per repeat unit is associated with capsule size, resistance to neutrophil-mediated killing, and average serotype carriage frequency [17],[44],[64]. Post-PCV7 prevalence data (2004) was obtained from Hanage et al [44]. Spearman correlation coefficients are shown, ^†^p<0.05, ^††^p<0.001.

**Figure 4:**
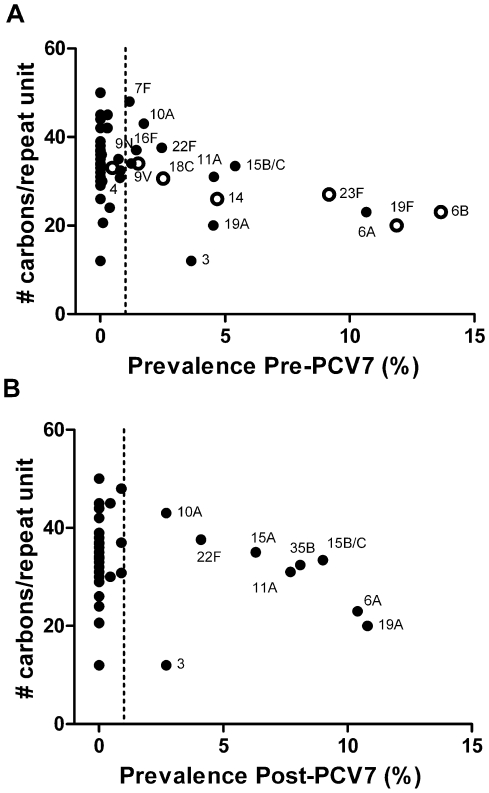
Relationship between capsule structure (carbon/repeat unit) and carriage prevalence [44]. Capsule structure is associated with prevalence both A) before introduction of conjugate vaccine (ρ = −0.80, p<0.001, excluding type 3) and B) after introduction of vaccine (2004) among children in Massachusetts (ρ = −0.90, p<0.05, excluding type 3). Vaccine types are denoted by an open circle.

Next, we evaluated the relationship between these correlates of polysaccharide structure and carriage prevalence. We began by assessing prevalence in populations not exposed to PCV7. Using carriage data pooled from three studies in the United Kingdom, the United States, and The Netherlands, we found a statistically significant inverse correlation between metabolic cost and serotype frequency among circulating types (Table 1, Figure 4). These analyses were performed among serotypes with an average frequency of at least 1%. As Figure 4 shows, serotypes that were extremely rare (<1%) had a broad range of carbons per repeat unit, suggesting that polysaccharide structure is not the sole determinant of the success of a serotype, and in particular suggesting that a low metabolic cost is necessary, but not sufficient for high prevalence in unvaccinated populations. We noted that serotype 3 – which has the least costly repeat unit structure and is known for its mucoid colony phenotype that is attributed to extensive capsule production – was an outlier in this relationship, with relatively low carriage prevalence. While the reason for its outlier status is unclear, it is notable that type 3 polysaccharide can generate a relatively strong antibody response early in infancy [43], and this serotype is highly clonal. Without serotype 3, the relationships become much stronger (Table 1).

Finally, we evaluated the relationship between polysaccharide structure and prevalence in a population exposed to PCV7, which has opened an ecological niche for serotypes not included in the vaccine. To date, no principle has been identified to predict which non-vaccine serotypes would become most common in vaccinated populations. We hypothesized that metabolic cost might provide such a principle to predict which non-vaccine serotypes would become most common in vaccinated populations. We compared the prevalence of serotypes in a post-vaccine carriage sample in Massachusetts in 2004 [12],[44] to measures of metabolic cost. Similar to what was seen before vaccination, there was no distinctive pattern of metabolic cost among the serotypes with frequencies <1%, but among those at appreciable frequencies, there was a negative relationship between metabolic cost and prevalence (Figure 4B), albeit here not statistically significant (Table 1). Once again, serotype 3 was an extreme outlier, and exclusion of type 3 resulted in a strong association between metabolic cost and prevalence (Table 1). We also found that increased degree of encapsulation was itself associated with higher post-vaccine carriage prevalence among the serotypes that we tested (Figure S4).

## Discussion

Our data suggest a novel biological explanation for serotype patterns of pneumococcal carriage. If clearance of pneumococcus from the nasopharynx depends on T-cell-mediated immunity [28],[31], we would expect that serotypes that successfully avoid neutrophil-mediated killing will persist longer in the nasopharynx (thereby also obtaining more opportunities for transmission) and, as a result, be more prevalent in the population. Our results demonstrate a clear link between capsular polysaccharide structure and prevalence. We propose a model in which serotypes producing polysaccharides that are less metabolically costly will be more heavily encapsulated and thus avoid phagocytic clearance and persist in carriage.

It is possible, though, that innate immune effectors in addition to or instead of neutrophils could determine the serotype prevalence hierarchy. In particular, phagocytes other than neutrophils could play a role, and antimicrobial peptides [22], mucus-mediated clearance [23], complement, C-reactive protein [45], and antibodies directed against the capsule or other surface antigens could all affect clearance rates of different serotypes from the nasopharynx. Likewise, some of these factors could affect the rate of acquisition of new carriage episodes, which would also influence the prevalence of a serotype.

While interactions with the host likely play a large role in shaping serotype patterns, polysaccharide production itself could affect the fitness of the bacterium and thus its ability to compete with other serotypes during colonization. We did not observe any meaningful differences in growth rate between serotypes *in vitro*, but it is possible that under *in vivo* conditions, polysaccharide production causes a competitive disadvantage for some serotypes. It is also possible that our measures of “metabolic cost” do not reflect the true cost to the bacteria since the organisms could respond to environmental pressures by changing their carbon and energy utilization.

Our findings provide a simple method for ranking serotype prevalence. The currently available vaccine for infants targets seven clinically relevant serotypes, but alternative formulations, including 10- and 13-valent vaccines, are being explored for use in developing countries. Since the relationship between polysaccharide structure and serotype prevalence is observed in both vaccinated and unvaccinated populations, this could potentially be used as a tool for predicting patterns of serotype replacement in different settings.

It has long been known that many characteristics of pneumococcal epidemiology were associated with serotype. Here, using two sets of pneumococci that are isogenic except for their capsular serotypes, we have shown that an *in vitro* predictor of these epidemiologic traits –susceptibility to non-opsonic killing – is determined in large part by capsular type rather than bacterial genetic background; moreover, using multiple clinical isolates, we have shown that these *in vitro* properties of different serotypes are consistent across diverse genetic backgrounds. However, it is also clear from our data that these *in vitro* properties can vary within capsular serotypes, consistent with the possibility that noncapsular factors, such as bacterial adhesins, could affect the success of a strain during colonization and could subsequently influence patterns of serotype replacement. Certain clones have been particularly successful and have acquired multiple serotypes [46],[47], further supporting the notion that other genetic factors influence the fitness of a pneumococcal strain. Likewise, while the types that are present at appreciable frequencies show a negative relationship between prevalence and metabolic cost, there are many serotypes with low metabolic costs that remain rare, most notably serotype 3. This finding suggests that a metabolically inexpensive capsule is necessary, but not sufficient, for high prevalence (Figure 4).

The mechanistic explanation for the association between polysaccharide structure and degree of encapsulation remains to be elucidated. We evaluated a number of structural features including the number of particular monosaccharide units, number of N-acetylated sugars and number and proportion of hydrophobic residues. One possibility is that limited supplies of energy or carbon impose more stringent limits on the production of metabolically costly capsule subunits than on the production of less costly ones. Another possibility, not mutually exclusive, results from the fact that serotypes with less costly polysaccharide repeat units also tend to have a higher ratio of charged molecules per carbon. While capsular charge does not have a large direct effect on interactions with neutrophils, it can affect the 3-dimensional stability of the capsule and the degree of encapsulation of the bacteria [39],[48]. Hence, capsular types with higher ratios of charge to carbon may have physically larger and/or more inhibitory capsules than those with lower ratios. Clearly, this latter mechanism is not the entire explanation, because the neutral type 14 capsule is relatively inhibitory (near the median in the neutrophil-killing assay), but it may play a contributory role. Whatever the mechanism(s) involved, our experimental manipulations of polysaccharide production for a single serotype suggest that capsular size itself affects interactions with the host even when structure is held constant.

It has previously been noted that there is an inverse correlation between carriage prevalence and invasiveness [6], and our findings may also explain this phenomenon. Bacteria attached to the epithelium tend to be less heavily encapsulated than unattached pneumococci [49]. It has been suggested that direct interaction with epithelial cells can promote invasion into underlying tissue and blood, and capsule could disrupt this process [50],[51]. As a result, serotypes that avoid association with neutrophils might also interact less with the epithelium and be less likely to invade. In contrast, highly invasive serotypes, such as types 4 and 5, are efficiently killed by neutrophils, but they might interact more closely with the mucosal surface and be more likely to invade.

We have evaluated degree of encapsulation by measuring the zone of inhibition of FITC-Dextran by the capsule. While this method does not directly determine polysaccharide quantity, it does provide a physiologically relevant measure of capsule thickness, which could in turn affect accessibility of a number of surface structures that could be recognized by neutrophils and other immune effectors.

These findings raise several additional questions that should be addressed in future studies. From the population-biological perspective, a key question is: if metabolically costly capsules reduce the ability of pneumococci to persist in the nasopharynx, how do these capsular types persist in the population in the face of competition from the metabolically less costly, more common types? One possibility is that these types occupy a distinct ecological niche. Such a niche could be a direct consequence of these strains' less pronounced capsule (perhaps allowing more intimate association with the epithelium) or could be created by the existence of serotype-specific immunity in some hosts, which may be most prevalent against the most common types, providing an advantage to the less common types to which fewer hosts are naïve [29].

In summary, these results suggest that the epidemiologic phenomena of serotype-specific prevalence in carriage and possibly serotype replacement can be explained, in substantial part, by bacterial characteristics measurable *in vitro* and furthermore by biochemical properties of the capsules themselves. Previously, it had been observed that capsular types differ in their epidemiological properties [4],[6],[16],[17], but it was not clear to what extent these properties resulted from the nature of the capsule itself or from the genetic backgrounds with which particular types – especially the rare types, which tend to be highly clonal – are associated. The hypothesis that capsule itself causes these epidemiologic differences is strengthened by our findings that the biochemical properties of the capsule predict epidemiologic properties, and that mechanistic intermediates such as degree of encapsulation and resistance to neutrophil killing, could be altered by changing only the capsule.

## Methods

### Bacteria and cells

Blood was obtained from healthy volunteers according to a protocol approved by the Office of Human Research Administration at Harvard School of Public Health. Neutrophils were isolated using a Histopaque 10771, 11191 gradient (Sigma-Aldrich, St. Louis, MO) according to the manufacturer's instructions and used immediately.

Capsule-switch variants were constructed on the TIGR4 genetic background [52] and were backcrossed at least three times, except for serotypes 1 and 5, in which we could only accomplish one backcross. The TIGR4 parent strain was itself backcrossed as a control for the transformation procedure. The 603 capsular variants were constructed with the same method and were all backcrossed 3 times, except for type 6B, which was backcrossed once as a transformation control. Nasopharyngeal clinical carriage isolates (Table S2) were colony-purified prior to use. Strain 603^WT^ is a type 6B invasive disease isolate [53], 603^cap-^ was constructed by replacement of the capsule biosynthesis locus with the Janus cassette, and 603^cap-:6B^ was constructed by transforming 603cap- with genomic DNA from the parent strain, as described previously [52],[54]. All strains were grown in Todd Hewitt Broth with 0.5% yeast extract (THY) (BD, Franklin Lakes, NJ) at 37° with 5% CO_2_ unless otherwise noted. In some cases, strains were grown in a semi-defined minimal media [55] with 1000 U/mL catalase (MP Biomedicals, Solon, OH) and 10 mM sugar.

### Neutrophil surface killing assay

Neutrophil surface killing assays were performed as described previously [31]. Briefly, bacteria were grown to mid-log phase and frozen in THY/10% glycerol at −80°. On the day of the experiment, bacteria were thawed and diluted to 5×10^3^ CFU/mL in saline, and 10 µL of this suspension was spotted and allowed to dry at room temperature on trypticase soy agar with 5% defibrinated sheep blood (TSA II) (BD) with 10 replicates per plate. Twenty microliters of neutrophils (2×10^6^ cells/mL) were then overlaid, allowed to dry, and incubated overnight at 37° with 5% CO_2_. Percent survival was calculated by comparing killing of each strain to a duplicate control plate with no neutrophils. All experiments were repeated on several days with different frozen lots of bacteria. The overall killing efficiency varied between experiments, so we normalized the data by dividing percent survival for each serotype by percent survival of type 9N to obtain relative survival. The mean relative survival from at least two independent experiments is presented unless otherwise stated. For experiments comparing susceptibility to killing of isogenic capsule switch variants and clinical isolates, the serogroup mean survival was used.

### FITC labeling of bacteria

Bacteria were grown to mid-log phase, washed in Hanks balanced salt solution (HBSS) (Cellgro,Manassas, VA) with 1% BSA, resuspended in FITC (Calbiochem, La Jolla, CA) (0.5 mg/mL in HBSS/1%BSA) and incubated for 1 hour at 4°. The bacteria were then washed twice and frozen in HBSS/1%BSA with 10% glycerol. Staining was evaluated by flow cytometry to ensure equivalent staining between strains.

### Neutrophil association assay

To evaluate bacterial association with human neutrophils, we developed a protocol based in part on Lee et al. [56]. In a 24-well dish, we added 1 mL of blood agar base (BD) per well and allowed it to solidify. FITC-labeled bacteria were then thawed, washed in HBSS/1%BSA, and resuspended to 1×10^8^ CFU/mL. 50 µL of bacteria were added to each well, allowed to dry, and overlaid with 2.5×10^5^ neutrophils in 50 µL. After 20 minutes, 1 mL ice cold HBSS/BSA was added to each well to harvest the neutrophils. This mixture was then washed twice and fixed with 1% formalin for 2 hours. Bacterial association with the neutrophils was assessed by flow cytometry using a MoFlo flow cytometer (Dako Cytomation, Denmark), and analysis was performed in Summit v4.3 (Dako). The average relative fluorescence from two independent experiments is presented.

### Capsule size determination

The degree of encapsulation was determined by measuring the zone of exclusion of FITC-dextran (2000 kDa, Sigma), based on the method of Gates et al. [39]. Bacteria were grown overnight on TSA II plates, swabbed into PBS, and 20 uL of bacteria were mixed with 2 uL of a 10 mg/mL stock solution of FITC-dextran, and used to create wet mounts with cover slips. The slides were viewed on a Nikon Eclipse 80i with a 100× objective, and fluorescent images were captured with a Spot RT SE camera. The images were analyzed with UTHSCSA ImageTool for Windows v3.0 (University of Texas Health Science Center in San Antonio). The area of FITC exclusion was determined, excluding chains and clumps of cells. For each serotype, the mean area of 100–250 cells was determined, and at least two images were collected from each of at least two independently prepared slides. The capsular zone was also visualized with India ink (Higgins, Oak Brook, IL) wet mounts, as described previously [57]. When visualized with this method, the bacterial body is surrounded by a bright halo, which results from light diffraction, and this halo is surrounded by a zone of clearance representing the capsule.

### Polysaccharide quantification

Strains were grown in 10 mL semi-defined media with 10 mM of glucose or fructose to mid-log phase and centrifuged. The concentration was adjusted to OD620 = 0.6 in 1 mL, and the suspension was lysed with 0.1% deoxycholate for 30 minutes and incubated with 100 U mutanolysin overnight at 37° [58]. Cell-associated type 19F capsular polysaccharide and phosphocholine were quantified using an inhibition ELISA, based on the method of Wessels et al [59]. Briefly, immulon-4 plates (Thermo Scientific, Waltham, MA) were coated by incubating overnight with 5 µg/mL cell wall polysaccharide (Statens Serum Institute, Copenhagen, Denmark) or 2 µg/mL type 19F capsular polysaccharide (ATCC, Manassas, VA). TEPC15 (Sigma, mouse monoclonal IgA directed against phosphocholine, 1:5000) or typing serum 19b (Statens, 1:10,000) was mixed 1:1 with bacterial lysates or standard dilutions of type 19F polysaccharide or cell wall, as appropriate. TEPC15 was detected with goat anti-mouse IgA-HRP (Southern Biotech, Birmingham, AL), and typing sera was detected with goat anti-rabbit IgG-HRP (Southern Biotech) (1:8000). After developing with TMB substrate (KPL, Gaithersburg, MD) and addition of 1N HCl, the absorbance at 450nm was determined.

### Animal infections

5 week old C57/BL6J (Jackson Laboratories, Bar Harbor, ME) were inoculated intranasally as described previously [31]. Briefly, mice were challenged with 3.5×10^6^ CFU of strain TIGR4:14 and 3.5×10^4^ CFU of strain TIGR4:19F. At one week post-inoculation, mice were euthanized by CO_2_ inhalation and tracheal washes were collected from the nostrils. Colonization density of each strain was determined by colony morphology and serotype was confirmed by latex agglutination (Miravista Diagnostics, Indianapolis, IN). All animals were handled in strict accordance with good animal practice, and all animal work was approved by the Harvard Institutional Animal Care and Use Committee.

### Biochemical information

Chemical structures of the capsular polysaccharide units were obtained from Kamerling [60]. Structural data was not available for serotypes 23A, 23B, 35F or 38, so they were excluded from the analysis. Pathways for central metabolism were obtained from www.biocyc.org [61]. Capsule-specific sugar precursor biosynthesis pathways were obtained from Bentley et al [62] and Aanensen et al [63]. For each serotype, we calculated the number of high-energy bonds (ATP, UTP, CTP, TTP) and the number of carbons required to generate each of the sugar precursors and subsequently the number of carbons and high-energy bonds required to generate one polysaccharide repeat unit (Table S1). We included high energy bonds required for importing carbon and glutamine and for converting monosaccharides to NDP-sugars. For energy calculations, we ignored acetate and pyruvate, since they are byproducts of normal metabolism.

### Statistics

Carriage prevalence data were obtained from [12],[17],[44],[64]. We calculated serotype frequency among carriage isolates and averaged among the three studies to minimize the effect of microepidemics. Only serotypes with frequency greater than 1% among carriage isolates were included in correlations between prevalence and biochemical properties.

Percent survival between strains was compared using either t-tests or ANOVA, as appropriate. Non-parametric Spearman correlation was used to evaluate the relationship between epidemiologic measures and resistance to neutrophil mediated killing or chemical composition of the capsule. For post-PCV7 correlations, serotypes targeted by the vaccine and those lacking structural information were ignored. Linear regression was used to evaluate the relationship between neutrophil-mediated killing of isogenic capsule switch variants and clinical carriage isolates. Analyses and graphing were performed in Graphpad Prism v5.0.

## Supporting Information

Figure S1 Relationship between carriage prevalence and resistance to neutrophil-mediated killing using isogenic capsule-switch variants constructed in strain 603.
(0.03 MB PDF)

Figure S2 Representative images of TIGR4:19F, TIGR4:5 suspended in India ink.
(0.05 MB PDF)

Figure S3 Growth of TIGR4 isogenic capsule-switch variants in fructose leads to increased susceptibility of heavily encapsulated serotypes to neutrophil-mediated killing compared to the same strains grown in glucose.
(0.04 MB PDF)

Figure S4 Relationship between serotype prevalence after vaccination in Massachusetts (2004) and A) degree of encapsulation (ρ = 0.85, p<0.01), and B) survival from neutrophil-mediated killing (ρ = 0.63, n.s.).
(0.04 MB PDF)

Table S1 Number of carbons and high energy bonds required to generate one polysaccharide repeat unit. For calculations of energy/repeat unit, acetate and pyruvate were excluded because they are byproducts of central metabolism. For calculations of carbon/repeat unit, choline was also excluded since it is imported into the cell and does not affect carbon utilization. Non-whole numbers are due to non-stoichiometric acetylation or addition of choline.
(0.05 MB PDF)

Table S2 Clinical isolates used in this study. Within a serogroup, isolates of diverse multi-locus sequence types were chosen when available.
(0.05 MB PDF)
