# Supplementary figures and images for: Pneumococcal Capsular Polysaccharide Structure Predicts Serotype Prevalence
Source: PLoS Pathog. 2009 Jun 12;5(6):e1000476. doi: 10.1371/journal.ppat.1000476 (PMC2689349; doi:10.1371/journal.ppat.1000476)

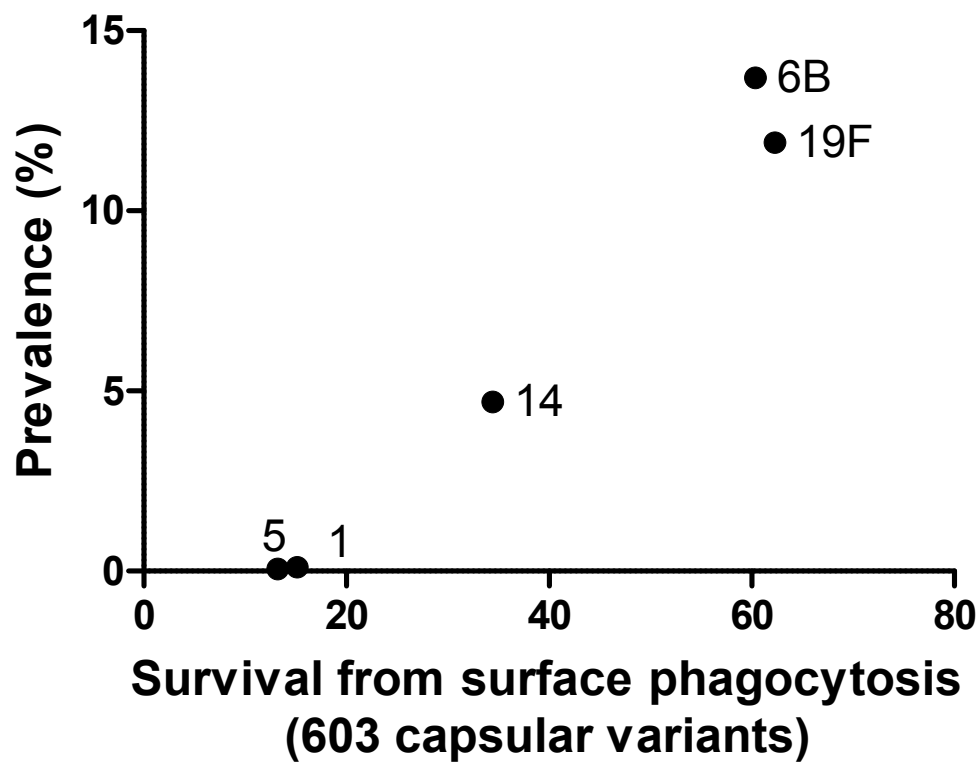

Supplement: Figure S1 — Relationship between carriage prevalence and resistance to neutrophil-mediated killing using isogenic capsule-switch variants constructed in strain 603. (0.03 MB PDF) [file ppat.1000476.s001.pdf]

Figure S2

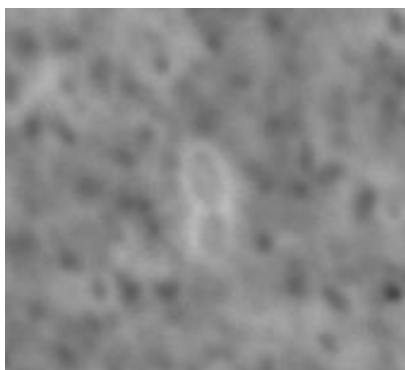

TIGR4:5

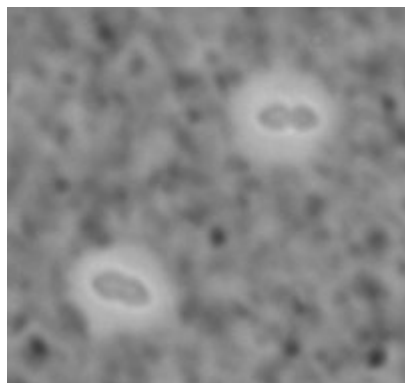

TIGR4:19F

Supplement: Figure S2 — Representative images of TIGR4:19F, TIGR4:5 suspended in India ink. (0.05 MB PDF) [file ppat.1000476.s002.pdf]

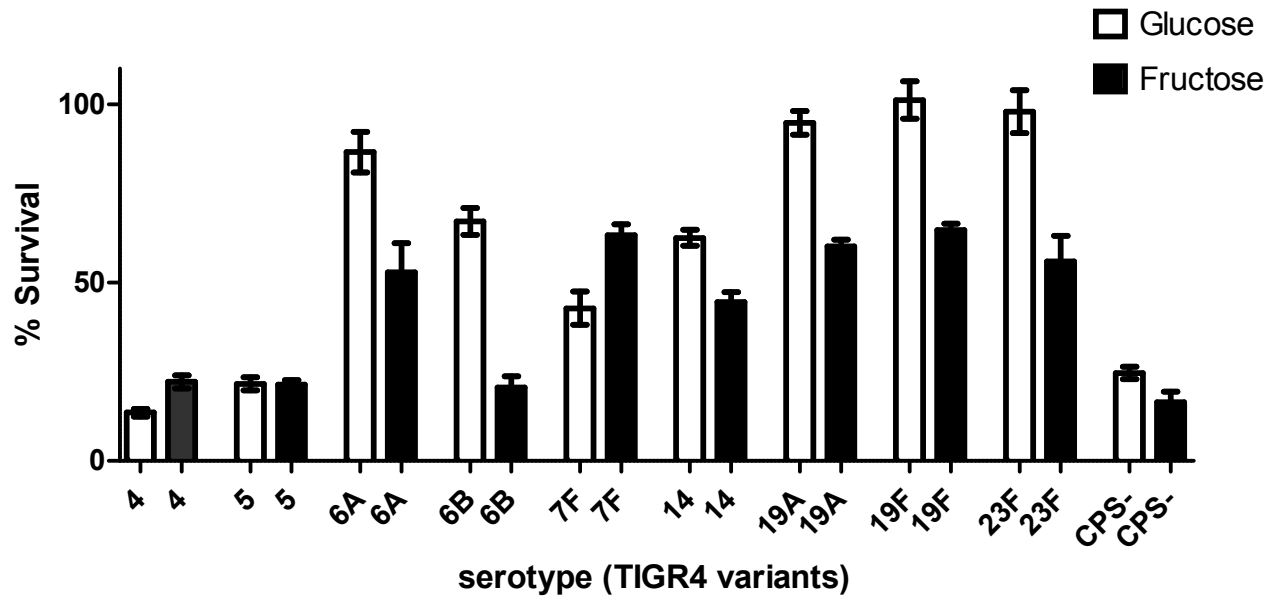

Supplement: Figure S3 — Growth of TIGR4 isogenic capsule-switch variants in fructose leads to increased susceptibility of heavily encapsulated serotypes to neutrophil-mediated killing compared to the same strains grown in glucose. (0.04 MB PDF) [file ppat.1000476.s003.pdf]

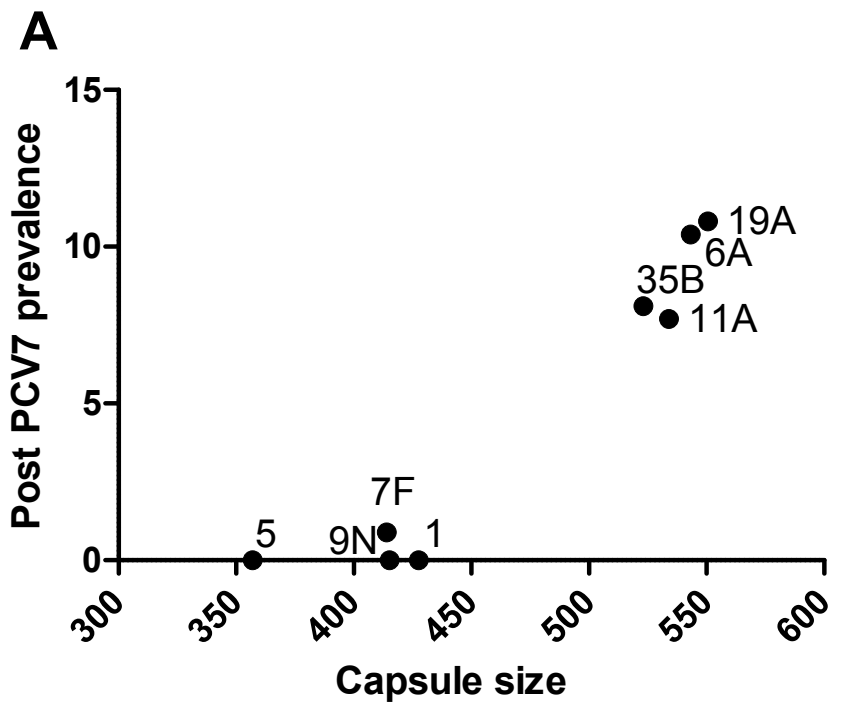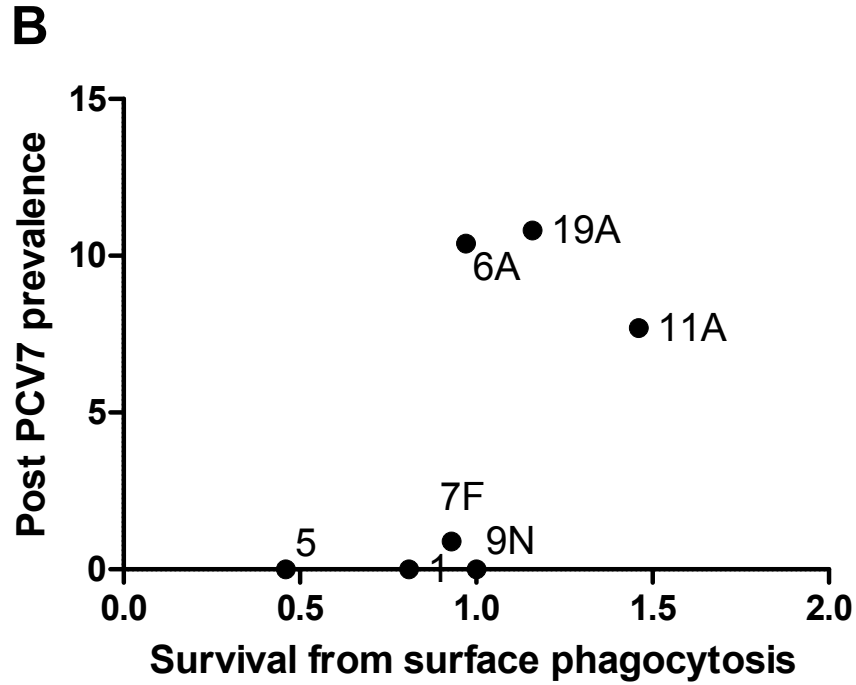

Supplement: Figure S4 — Relationship between serotype prevalence after vaccination in Massachusetts (2004) and A) degree of encapsulation (ρ = 0.85, p<0.01), and B) survival from neutrophil-mediated killing (ρ = 0.63, n.s.). (0.04 MB PDF) [file ppat.1000476.s004.pdf]
