# Supplementary material for: Pneumococcal Capsular Polysaccharide Structure Predicts Serotype Prevalence
Source: PLoS Pathog. 2009 Jun 12;5(6):e1000476. doi: 10.1371/journal.ppat.1000476 (PMC2689349; doi:10.1371/journal.ppat.1000476)
Supplement: Table S1 — Number of carbons and high energy bonds required to generate one polysaccharide repeat unit. For calculations of energy/repeat unit, acetate and pyruvate were excluded because they are byproducts of central metabolism. For calculations of carbon/repeat unit, choline was also excluded since it is imported into the cell and does not affect carbon utilization. Non-whole numbers are due to non-stoichiometric acetylation or addition of choline. (0.05 MB PDF) [file ppat.1000476.s005.pdf]

Table S1. Number of carbons and high energy bonds required to generate one polysaccharide repeat unit. For calculations of energy/repeat unit, acetate and pyruvate were excluded because they are byproducts of central metabolism. For calculations of carbon/repeat unit, choline was excluded since it is imported into the cell and does not affect carbon utilization. Non-whole numbers are due to non-stoichiometric acetylation or addition of choline.

Serotype	Carbon	High Energy	Serotype	Carbon	High Energy
1	20.6	8	17A	50	16
2	36	12	17F	45	14
3	12	4	18A	35	13
4	33	11	18B	33	12
5	36	13	18C	30.6	10
6A	23	8	18F	37	12
6B	23	8	19A	20	7
7A	42	14	19B	34	14
7B	38	15	19C	34	14
7F	48	16	19F	20	7
8	24	8	20	42	13
9A	32	11	22F	37.6	12
9L	34	12	23A	na	na
9N	34	12	23B	na	na
9V	34	11	23F	27	10
10A	43	15	27	29	11
10F	37	13	29	31	11
11A	31	10	31	30	10
11B	33.8	11	32A	26	10
11C	31	11	32F	26	10
11F	35	11	33B	39	13
12A	44	16	33F	36.8	12
12F	42	15	34	30	10
13	33	11	35A	30	10
14	26	9	35B	32.4	11
15A	35	13	35F	na	na
15B	33.4	11.4	37	12	4
15C	32	11.4	45	45	17
16F	37	14			
