# Supplementary material for: Pneumococcal Capsular Polysaccharide Structure Predicts Serotype Prevalence
Source: PLoS Pathog. 2009 Jun 12;5(6):e1000476. doi: 10.1371/journal.ppat.1000476 (PMC2689349; doi:10.1371/journal.ppat.1000476)
Supplement: Table S2 — Clinical isolates used in this study. Within a serogroup, isolates of diverse multi-locus sequence types were chosen when available. (0.05 MB PDF) [file ppat.1000476.s006.pdf]

Table S2. Clinical isolates used in this study. Within a serogroup, isolates of diverse multi-locus sequence types were chosen when available.

Serotype	Isolates tested (#)	Multi-locus sequence types	Site of Isolation
1	3	227, unknown (2)	Arizona, Israel
4	3	205, 247, unknown	Arizona, Israel
5	3	Unknown	Israel
6A	1	3034	Arizona
6B	1	3075	Arizona
7C	3	1757 (2), unknown (1)	Arizona, Israel
9L	1	3061	Arizona
9N	1	Unknown	Israel
9V	1	156	Arizona
14	4	124 (2), 782, 2194	Arizona
18C	3	113, unknown (2)	Arizona, Israel
19A	2	199	Arizona
19F	2	177, 1258	Arizona
23A	1	1448	Arizona
23B	1	62	Arizona
23F	1	33	Arizona
